# Primary care challenges in diagnosing and referring patients with suspected rheumatoid arthritis: a national cross-sectional GP survey

**DOI:** 10.1093/rap/rky012

**Published:** 2018-04-06

**Authors:** Ian C Scott, Navjeet Mangat, Alex MacGregor, Karim Raza, Christian D Mallen, Samantha L Hider

**Affiliations:** 1 Arthritis Research UK Primary Care Centre, Research Institute for Primary Care & Health Sciences, Primary Care Sciences, Keele University, Newcastle-under-Lyme; 2 Department of Rheumatology, Haywood Hospital, High Lane, Burslem, Staffordshire; 3 Department of Medical and Molecular Genetics, King’s College London, Guy’s Hospital, Great Maze Pond, London; 4 Norwich Medical School, University of East Anglia, Norwich; 5 Institute of Inflammation and Ageing, College of Medical and Dental Sciences, University of Birmingham, Edgbaston, Birmingham; 6 Sandwell and West Birmingham Hospital NHS Trust, Birmingham, UK

**Keywords:** rheumatoid arthritis, referral, primary care, guidelines

## Abstract

**Objective:**

National guidelines advocate referring patients with persistent synovitis to rheumatology within 3 working days of presentation to primary care. This occurs infrequently. We aimed to identify modifiable barriers to early referral of suspected RA patients among English general practitioners (GPs).

**Methods:**

We carried out a national cross-sectional survey of 1388 English GPs (RA Questionnaire for GPs [RA-QUEST] study). Questions addressed GPs’ confidence in diagnosing RA, clinical factors influencing RA diagnosis/referral, timeliness of referrals and secondary care access. Data were captured using 10-point visual analog scales, five-point Likert scales, yes/no questions or free text, and were analysed descriptively.

**Results:**

Small joint swelling and pain were most influential in diagnosing RA (91 and 84% rated the importance of these as 4 or 5 on a five-point Likert scale, respectively); investigations including RF (61% rating 4 or 5) and anti-CCP antibody (72% rating 4 or 5) were less influential. Patient history had the greatest impact on the decision to refer (92% rating this 4 or 5 on a 5-point Likert scale), with acute phase markers (74% rating 4 or 5) and serology (76% rating 4 or 5) less impactful. Despite the importance placed on history and examination, only 26% referred suspected RA immediately without investigations; 95% of GPs organizing further tests opted to test for RF.

**Conclusion:**

For suspected RA patients to be referred within 3 days of presentation to primary care there needs to be a paradigm shift in GPs’ approaches to making referral decisions, with a focus on clinical history and examination findings, and not the use of investigations such as RF.

Key messages

- Most general practitioners organize tests before deciding to refer suspected RA patients.
- An over-reliance is placed on RF testing when making referral decisions for suspected RA.
- A change in referral practice is required, making decisions based on clinical findings.

## Introduction

The early diagnosis and prompt treatment of RA by specialists improves patient outcomes [1]. In England, the National Institute for Health and Care Excellence (NICE) Quality Standards for RA recommend that patients with persistent synovitis are referred to a rheumatology service within 3 working days of presentation to primary care [2]. The British Society for Rheumatology Healthcare Quality Improvement Partnership national audits based on these quality standards highlighted the challenges in achieving them [3], with only 17 and 20% of patients referred within 3 working days, in the first and second audits, respectively. Similar referral delays from primary to secondary care exist in other European countries [4] and North America [5].

Several factors contribute to these referral delays. Firstly, the rarity of RA (annual incidence 15/100 000 adults [3]) means that non-specialists lack experience in recognizing it. Secondly, the heterogeneous nature of early RA can make identifying it challenging [6, 7]. Thirdly, general practitioners (GPs) traditionally make diagnoses before referral, using investigations to support their clinical opinion; requesting tests in patients with suspected RA will invariably delay the referral process.

Variations in national health-care structures mean that factors contributing to referral delays need to be considered on a country-specific basis. Data on factors associated with GP referral delays of suspected RA in England are limited, but existing studies suggest that referral decisions are strongly influenced by test results (chiefly RF and radiographs), with negative/normal tests making referral less likely or timely [8–10]. These studies are limited by their regional nature [10], small size [8] or focus on a single factor [9].

To increase the proportion of RA referrals meeting the NICE quality standard time line (3 working days) a range of modifiable barriers to early referral need to be identified, which have generalizable impacts across England. The RA Questionnaire for GPs (RA-QUEST) study was designed to achieve this. It is a large, prospective survey of 1388 English GPs’ experiences in diagnosing and referring suspected RA patients to secondary care.

## Methods

### National GP survey

Five thousand English GPs, randomly selected using Binley’s database (National database of GP practice contact details) [11], were mailed a questionnaire in 2014, asking 12 questions about challenges in diagnosing and referring suspected RA patients, alongside questions about their demographics and primary care practice.

### Questionnaire development

The questionnaire was developed by a focus group of clinical and academic GPs, and rheumatologists at Keele University; it was subsequently piloted and refined with local GPs before national implementation. Question items were sought to cover GP access to rheumatology, knowledge of RA symptoms/signs, confidence in diagnosing RA and which factors influence the decision to refer and time scale of referral.

### Questions about challenges in diagnosing and referring suspected RA

The 12 questions about diagnosing and referring patients with suspected RA are provided in supplementary Table S1 and Fig. S1, available at *Rheumatology Advances in Practice* online. In brief, they evaluated GP confidence in diagnosing RA and recognizing synovitis; how many patients GPs suspected they had seen with new-onset RA in the previous 2 years; what GPs felt were the most important symptoms in diagnosing RA (with the symptoms listed being derived from a previous qualitative study of symptom complexes during the earliest phases of RA [7]); if they had heard of the S-factor campaign (an Arthritis Research UK/National RA Society delivered campaign promoting the need for patients to consult their GP early for symptoms of RA [12]) and its impact on their practice; what they felt were the most important features in making a decision to refer a patient with suspected RA; whether they referred patients with suspected RA immediately or requested further tests first; their access to secondary care rheumatology; and what they felt were the challenges in making an RA diagnosis. These questions were completed using a mixture of: (i) 10-point visual analog scales (VAS), for example, ‘How confident are you at diagnosing RA?’ on a scale of 0 (not at all confident) to 10 (completely confident); (ii) yes/no responses, for example, ‘Do you have access to a dedicated early arthritis clinic?’; (iii) five-point Likert scales; or (iv) free-text boxes.

### Statistical analysis

All data were summarized descriptively, using mean (s.d.), median [interquartile range (IQR)] and number (percentage) where appropriate based on data type, and distributions. The associations between GP time since qualification and gender, and confidence in diagnosing RA and referral practice, were evaluated using linear and logistic regression models. Missing data were omitted from the analysis (supplementary Table S2, available at *Rheumatology Advances in Practice* online).

### Ethical approval

The study was approved by the Keele University Ethics Review Panel. As it represented an anonymous study of primary care practitioners, national ethical committee approval was not required. Written informed consent was obtained from participating practitioners.

## Results

### General practitioner characteristics

One thousand three hundred and eighty-eight completed questionnaires were returned (28% response rate). Most GPs were partners (845; 61%), with salaried (291; 21%), senior partner (207; 15%) and locum (36; 3%) GPs being less common. Their mean age was 47 years, mean time since qualification was 23 years, and 705 (51%) were male. Only 38 GPs (3%) had heard of the S-factor campaign. Of those completing the free-text response regarding its impact on their clinical practice, the commonest responses were that it helped in identifying patients with RA (nine GPs; 24%), increased awareness of RA (four GPs; 11%), meant they were more likely to refer suspected RA patients early (three GPs; 8%) or had no impact (nine GPs; 24%). A bar-plot outlining these responses is given in supplementary Fig. S2, available at *Rheumatology Advances in Practice* online. The median (IQR) score for the number of patients with suspected RA seen over the preceding 2 years was 4 (2–6).

### Access to rheumatology

**Fig. 1:**
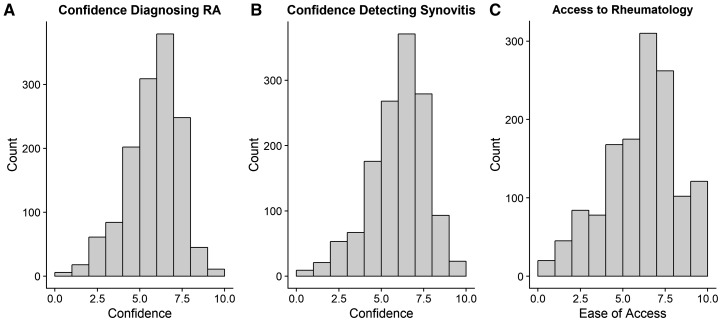
Confidence in diagnosing RA (A) and detecting synovitis (B), and ease of access to rheumatology (C) (**A**) General practitioner (GP) confidence on Visual Analogue Scale (0=not at all confident; 10=completely confident) in diagnosing RA. (**B**) GP confidence on Visual Analogue Scale (0=not at all confident; 10=completely confident) in recognizing synovitis. (**C**) GP rating ‘How easy is it for you to access secondary care rheumatology?’ on a visual analog scale (0=very difficult; 10=very easy).

Four hundred and ninety-eight (38%) GPs had access to dedicated early arthritis clinics. The median (IQR) VAS rating for ease of access to secondary care rheumatology was 7 (5–8), indicating that most GPs considered they had moderate ease of access (Fig. 1). General practitioners reporting access to dedicated early arthritis clinics had a higher median (IQR) VAS (7; 6–8) for ease of access to rheumatology compared with those reporting no access to early arthritis clinics (6; 5–8).

### Challenges in diagnosing RA

#### Key clinical features

**Fig. 2:**
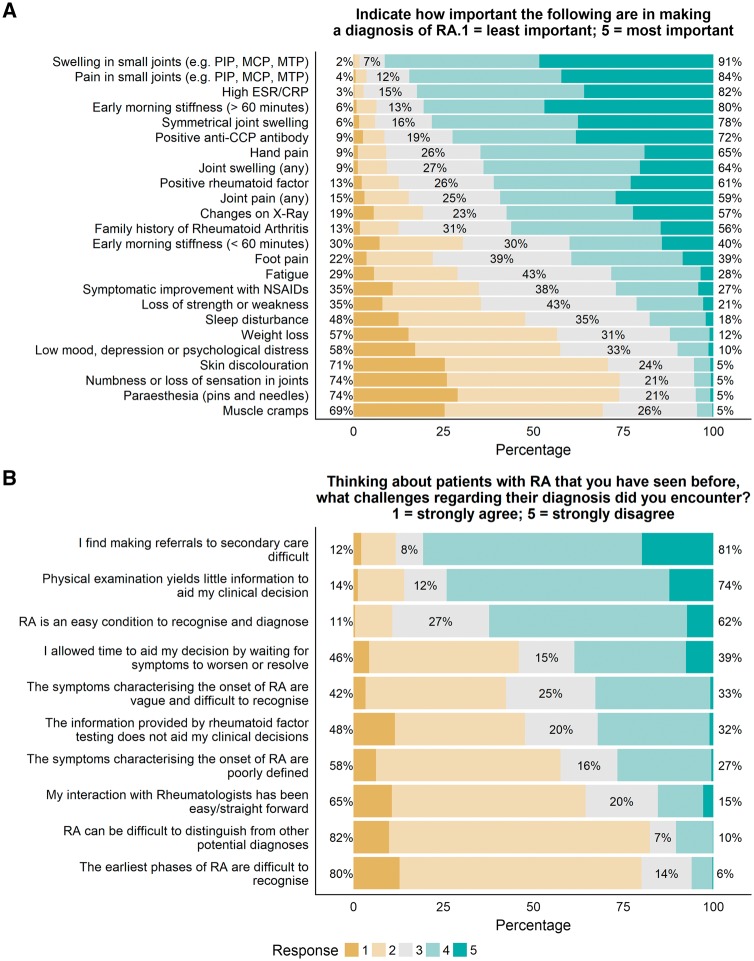
GP responses to Likert scale questions on the important clinical features (**A**) and perceived challenges (**B**) in diagnosing RA.

Of the 24 clinical features provided, GPs identified the following five as the most important in diagnosing RA (Fig. 2): small joint swelling (91% rated this 4 or 5 for importance, out of a possible 5), small joint pain (84% rated this 4 or 5 for importance), raised ESR/CRP (82% rated this 4 or 5 for importance), early morning stiffness >60 min (80% rated this 4 or 5 for importance) and symmetrical joint swelling (78% rated this 4 or 5 for importance). Median (IQR) Likert scores were 4 (4–5) for all five features.

Likert scores for other features included in RA classification criteria [13, 14] were considered less diagnostically important: positive anti-CCP (72% rated this 4 or 5 for importance), any joint swelling (64% rated this 4 or 5 for importance), positive RF (61% rated this 4 or 5 for importance) and radiographic changes consistent with RA (57% rated this 4 or 5 for importance). Median (IQR) Likert scores were 4 (3–5) for anti-CCP and 4 (3–4) for the other clinical features.

#### Confidence

General practitioners were moderately confident at diagnosing RA and detecting synovitis, with median self-rated VAS of 7 (5–7) and 7 (6–8) out of 10, respectively (Fig. 1).

#### Key challenges

The main perceived challenges in diagnosing RA were ‘the earliest phases of RA are difficult to recognize’, and ‘RA can be difficult to distinguish from other potential diagnoses’, with 80 and 82% strongly/moderately agreeing with these statements, respectively (Fig. 2). Despite often requesting RF before making a decision to refer, 48% strongly/moderately agreed with the statement ‘Information provided by RF testing does not aid my clinical decisions’. Two hundred and forty-four GPs provided free-text information in response to question 12 (addressing the challenges faced by GPs in diagnosing RA), with the main challenge being a perceived delay in access to secondary care services (reported by 98 GPs; 40.2%; supplementary Fig. S2, available at *Rheumatology Advances in Practice* online).

### Referral decisions

#### Factors influencing referrals

General practitioners rated patient history as the most important clinical feature in making a decision to refer, with 92% rating this 4 or 5 [median (IQR) score 5; 4–5] out of a possible 5 (supplementary Fig. S3, available at *Rheumatology Advances in Practice* online). Similar Likert scores were obtained for clinical examination [81% rating 4 or 5; median (IQR) score 4; 4–5], RF/anti-CCP serology [76% rating 4 or 5; median (IQR) score 4; 4–5] and raised ESR/CRP [74% rating 4 or 5; median (IQR) score 4; 3–5]. Little weight was placed on family history of RA [39% rating 4 or 5; median (IQR) score 3; 3–4]. Seventy-eight GPs provided free-text information on additional factors they felt important in making a decision to refer a patient (supplementary Fig. S2, available at *Rheumatology Advances in Practice* online), with the commonest responses being X-rays (14 GPs; 17.9%), disability (seven GPs; 9%), persistent or severe symptoms (seven GPs; 9%), stiffness (seven GPs; 9%) and synovitis (seven GPs; 9%).

#### Referral timeliness

Only 343 (26%) GPs would refer suspected RA immediately to secondary care; 999 (74%) preferred to organize further tests to inform referral decisions. Of the GPs who would organize further tests, the most frequently requested were RF (944 GPs; 95%), CRP (932 GPs; 93%) and ESR (883 GPs; 88%). Radiographs (544 GPs; 55%) and anti-CCP antibody testing (433 GPs; 43%) were less commonly used, and joint US (32 GPs; 3%) was used rarely. One hundred and sixty GPs provided free-text information on additional tests they would use (supplementary Fig. S2, available at *Rheumatology Advances in Practice* online), with the commonest being a list of multiple different blood tests (many of which included ANA and uric acid; 75 GPs; 46.9%), ANA and other autoantibodies (19 GPs; 11.9%) and full blood count tests (17 GPs; 10.6%).

### Associations between GP demographics, confidence and referral practice

#### General practitioner time since qualification

In a linear regression model, which included confidence in diagnosing RA (on a 10-point VAS) as the response variable and time since qualification (in years) as the explanatory variable, a significant association was observed (*P *=* *0.01), suggesting that GP confidence at diagnosing RA increases as more clinical experience is accrued. The effect was, however, small, with a β-value of 0.01, indicating that per 10-year increase in the time since qualification, the confidence in diagnosing RA VAS increased by merely 0.10 (out of a possible 10 units).

In a logistic regression model including the binary answer to the question, ‘If you suspect RA clinically do you refer immediately or arrange further tests first?’ as the response variable and time since qualification as the explanatory variable, no association was seen (*P *=* *0.62).

#### General practitioner gender

Undertaking the same modelling approach but including GP gender as the explanatory variable (in place of time since qualification), an association was observed between gender and reported confidence in diagnosing RA (*P *<* *0.01) but not referral practice (*P *=* *0.49). Female GPs appeared to be more confident at diagnosing RA. The β-value of 0.45 obtained from the linear regression model indicated that females had a 0.45 higher VAS for confidence in diagnosing RA than males.

## Discussion

Our national survey of English GPs found that when they suspect a patient has RA, the majority (74%) request investigations to support their clinical opinion before referral. Consequently, most GPs cannot meet the NICE quality standard of referring patients with persistent synovitis within 3 days. Meeting this quality standard requires a paradigm shift in the primary care approach to inflammatory arthritis referrals, with patients presenting with synovitis being referred on clinical grounds without waiting for the results of investigations. As our survey showed that GPs have a good knowledge of the clinical features of RA (with most correctly identifying small joint swelling, pain, early morning stiffness and symmetrical joint swelling as the most important symptoms/signs) this change in practice should be achievable.

We found an over-reliance on RF testing in primary care, undertaken by 95% of those GPs requesting tests before referral. Although we did not capture information on whether RF status influences final referral decisions, two previous English studies reported that RF-negative patients were less likely to be referred [10] or were referred significantly later [9]. Another study of 36 191 RF requests made to one English laboratory between 2003 and 2009, at an annual cost of £58 164, found that the majority (67%) originated from primary care, with only 7% made by rheumatologists [15]. The rate of positive results in primary care was low, at 6%, compared with 18% for rheumatologists. When these findings are considered against NICE recommendations, there is an argument for restricting the use of RF testing to rheumatology units.

Another major source of delay in suspected RA patients being seen lies with secondary care services failing to see primary care referrals promptly. Our study suggests that this is an ongoing issue, with 62% of GPs reporting no access to early arthritis clinics, and 25% rating their ease of access to rheumatology as being ≤5 out of 10. The need to minimize secondary care delay is also addressed in the NICE RA Quality Standards, which recommend that people with suspected persistent synovitis are assessed in a rheumatology service within 3 weeks of referral. The British Society for Rheumatology Healthcare Quality Improvement Partnership audit reported that the presence of early inflammatory arthritis clinics increased the odds of meeting this standard by 60% (OR = 1.6; 95% CI: 1.4, 1.7; *P *<* *0.001). This suggests that changes in primary care referral practice need to be linked with an increased provision of early inflammatory arthritis clinics.

Our study’s strength is that it represents a large national survey, with GP practices randomly selected. Its limitation is the modest response rate (28%). Our response rate is, however, similar to other recent national UK surveys [16, 17], and a low response rate does not necessarily indicate non-response bias [18], with previous research showing similar results in early survey responders compared with those responding after intensive contact attempts [19].

In conclusion, our findings suggest that to increase the proportion of suspected RA patients being referred within 3 days of presentation to primary care, there needs to be a paradigm shift in GPs’ approaches to making referral decisions in patients with synovitis, moving away from the use of investigations to confirm their clinical suspicion of RA and towards referring patients based on clinical findings. Further research is required to determine the best manner to implement this change in referral practice and evaluate its impact on attaining NICE quality standards.

*Funding*: This paper presents independent research funded by the National Institute for Health Research (NIHR) and Arthritis Research UK. It was supported by the Arthritis Research UK Centre in Primary Care grant (Grant Number 18139). C.D.M. is funded by the NIHR Collaborations for Leadership in Applied Health Research and Care West Midlands, the NIHR School for Primary Care Research and an NIHR Research Professorship in General Practice (NIHR-RP-2014-04-026). K.R. is supported by the Birmingham NIHR Biomedical Research Centre. The views expressed are those of the author(s) and not necessarily those of the National Health Service, the NIHR or the Department of Health and Social Care.

*Disclosure statement*: K.R. has received an educational grant from Abbvie and has received honoraria from Janssen, Pfizer and Roche. The other authors declare no relevant conflicts of interest.

## Supplementary Material

Supplementary Data
