# Supplementary material for: Primary care challenges in diagnosing and referring patients with suspected rheumatoid arthritis: a national cross-sectional GP survey
Source: Rheumatol Adv Pract. 2018 Apr 6;2(1):rky012. doi: 10.1093/rap/rky012 (PMC6597052; doi:10.1093/rap/rky012)

**Supplementary Table 1. Questions About Challenges in Diagnosing and Referring Patients with Suspected Rheumatoid Arthritis**

| **Question** | **Scoring** |
| --- | --- |
| 1. How confident are you at diagnosing RA? | 10-point VAS  (0=not at all confident; 10=completely confident) |
| 2. How confident are you in recognising joint swelling due to synovitis? | 10-point VAS  (0=not at all confident; 10=completely confident) |
| 3. How many patients have you suspected have new RA in the last 2 years? | Free-text box |
| 4. Please indicate how important the following symptoms are in making a diagnosis of RA: *a. Joint pain (any); b. Hand pain; c. Foot pain; d. Pain in small joints; e. Early morning stiffness <60 minutes; f. Early morning stiffness >60 minutes; g. Symptomatic improvement with NSAIDs; h. Family history of RA; i. Joint swelling (any); j. Swelling in small joints; k. Symmetrical joint swelling; l. High ESR/CRP; m. Positive rheumatoid factor; n. positive anti-CCP antibody; o. Changes on X-ray; p. Fatigue; q. Loss of strength or weakness; r. Muscle cramps; s. Low mood, depression, or psychological distress; t. Weight loss; u. Sleep disturbance; v. Skin discolouration; w. Numbness or loss of sensation in joints; x. Paraesthesia.* | 5-point Likert scale for each of the items (from a to x)  (1=least important; 5=most important) |
| 5. Have you heard of the S factor campaign for RA? | Yes/no response |
| 6. If yes to question 5, how has this impacted on your clinical practice | Free-text box |
| 7. Please indicate how useful the following are when making a decision to refer a patient to a Rheumatologist: *a. Patient history; b. Clinical examination; c. Family history of RA; d. Raised ESR/CRP; e. Positive rheumatoid factor/anti-CCP antibody result; f. Other (please state)* | 5-point Likert scale for each of the items (a to f)  (1=least useful; 5=most useful) |
| 8. If you suspect RA clinically do you: *a. Refer immediately; b. Arrange further tests first* | Choose one response only |
| 9. If you arrange further tests, which tests do you request: *a. ESR; b. Rheumatoid factor; c. CRP; d. X-rays; e. Anti-CCP; f. Ultrasound; g. Other (please state)* | Choose all tests that apply |
| 10. Do you have access to a dedicated early arthritis clinic? | Yes/no response |
| 11. How easy is it for you to access secondary care rheumatology? | 10-point VAS (0=very difficult; 10=very easy) |
| 12. Thinking about patients with RA that you have seen before, what challenges regarding their diagnosis did you encounter?: *a. RA can be difficult to distinguish from other potential diagnoses; b. Symptoms characterising the onset of RA are poorly defined; c. Information provided by rheumatoid factor testing does not aid my clinical decisions; d. Physical examination yields little information to aid my clinical decision; e. Symptoms characterising the onset of RA are vague and difficult to recognise; f. I allowed time to aid my decision by waiting for symptoms to worsen or resolve; g. The earliest phases of RA are difficult to recognise; h. I find making referrals to secondary care difficult; i. RA is an easy condition to recognise and diagnose; j. My interaction with rheumatologists has been easy/straight forward; k. Other challenges (please state)* | 5-point Likert scale for each of the items (a to j)  (1=strongly agree; 5=strongly disagree) |

VAS=visual analogue scale

**Supplementary Table 2. Number of Items with Missing Data (Responses from 1,388 GPs)**

| **Question** | **Question Item** | **Number (%) Missing Data** |
| --- | --- | --- |
| Q1- confidence diagnosing RA | - | 25 (2%) |
| Q2- confidence detecting synovitis | - | 28 (2%) |
| Q3 - number seen with suspected RA | - | 347 (25%) |
| Q4- important symptoms diagnosing RA | a) joint pain | 52 (4%) |
|  | b) hand pain | 66 (5%) |
|  | c) foot pain | 77 (6%) |
|  | d) pain in small joints | 52 (4%) |
|  | e) EMS <60 mins | 90 (7%) |
|  | f) EMS >60 mins | 47 (3%) |
|  | g) improvement with NSAIDs | 67 (5%) |
|  | h) family history of RA | 56 (4%) |
|  | i) joint swelling | 68 (5%) |
|  | j) small joint swelling | 39 (3%) |
|  | k) symmetrical joint swelling | 56 (4%) |
|  | l) high ESR/CRP | 37 (3%) |
|  | m) positive RF | 40 (3%) |
|  | n) positive anti-CCP | 136 (10%) |
|  | o) X-ray changes | 50 (4%) |
|  | p) fatigue | 50 (4%) |
|  | q) loss of strength/weakness | 59 (4%) |
|  | r) muscle cramps | 58 (4%) |
|  | s) low mood | 58 (4%) |
|  | t) weight loss | 58 (4%) |
|  | u) sleep disturbance | 54 (4%) |
|  | v) skin discolouration | 57 (4%) |
|  | w) numbness | 59 (4%) |
|  | x) paraesthesia | 53 (4%) |
| Q5- heard of S factor campaign | - | 18 (1%) |
| Q7- utility in referral decision | a) history | 17 (1%) |
|  | b) examination | 20 (1%) |
|  | c) family history of RA | 25 (2%) |
|  | d) raised ESR/CRP | 19 (1%) |
|  | e) positive RF/anti-CCP | 25 (2%) |
| Q8- action if suspect RA clinically | a) refer immediately | 46 (3%) |
|  | b) arrange further tests | 46 (3%) |
| Q9- if further tests, what is requested* | a) ESR | 0 (0%) |
|  | b) RF | 0 (0%) |
|  | c) CRP | 0 (0%) |
|  | d) X-rays | 2 (<1%) |
|  | e) anti-CCP | 1 (<1%) |
|  | f) USS | 2 (<1%) |
| Q10- access to early arthritis clinic | - | 94 (7%) |
| Q11- access to rheumatology | - | 23 (2%) |
| Q12- challenges in diagnosing RA | a) difficult to distinguish from other diagnoses | 20 (1%) |
|  | b) poorly defined symptoms | 26 (2%) |
|  | c) RF unhelpful | 24 (2%) |
|  | d) examination unhelpful | 18 (1%) |
|  | e) symptoms vague | 20 (1%) |
|  | f) allow time to aid decision | 24 (2%) |
|  | g) earliest phases difficult to recognise | 19 (1%) |
|  | h) secondary care referrals difficult | 22 (2%) |
|  | i) RA is easy to recognise and diagnose | 22 (2%) |
|  | j) interaction with rheumatologists easy | 20 (1%) |

*this is the percentage of the 999 individuals requesting further tests before referral.

**Supplementary Figure 1. RA-QUEST GP Survey**

**
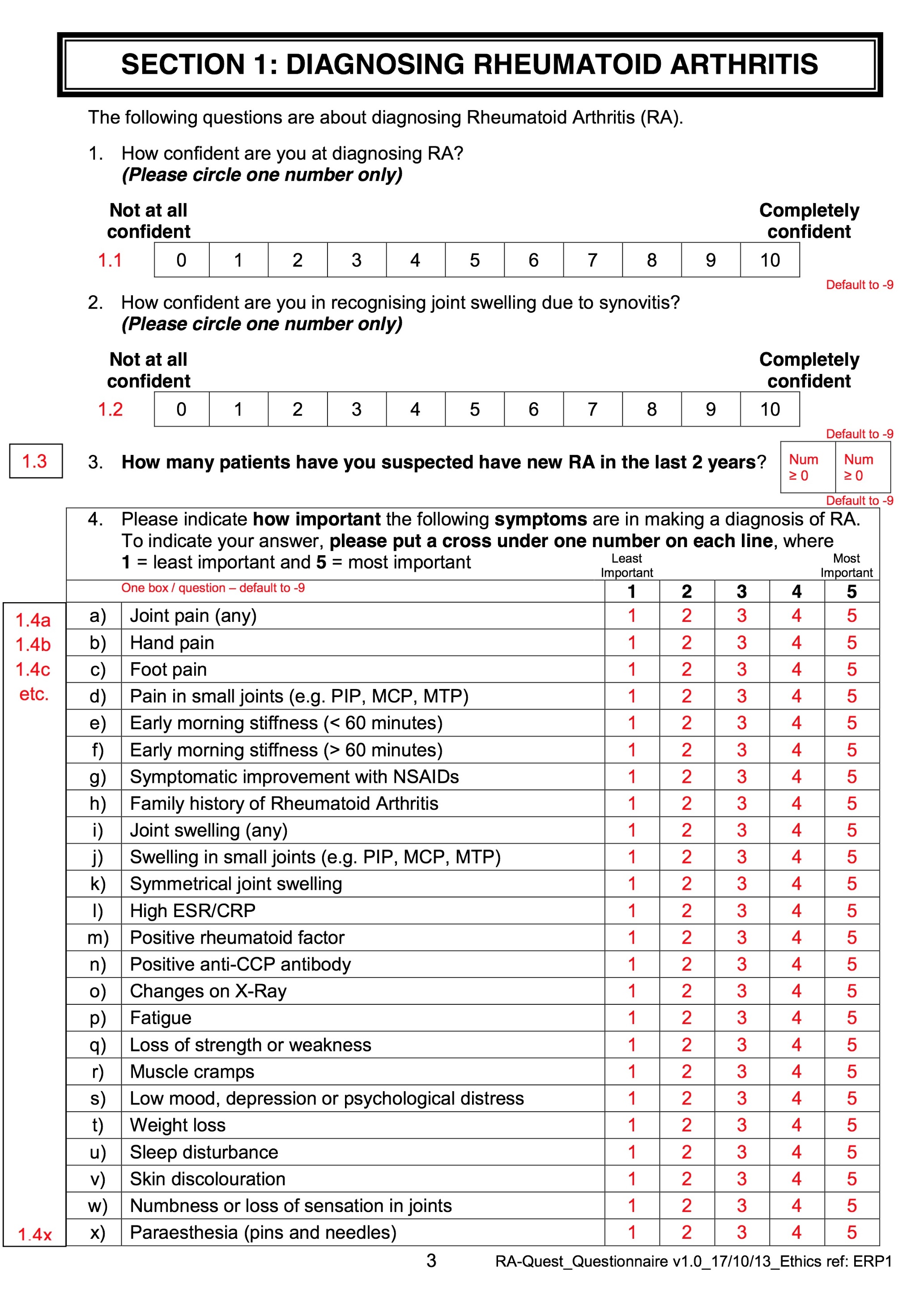
**

**
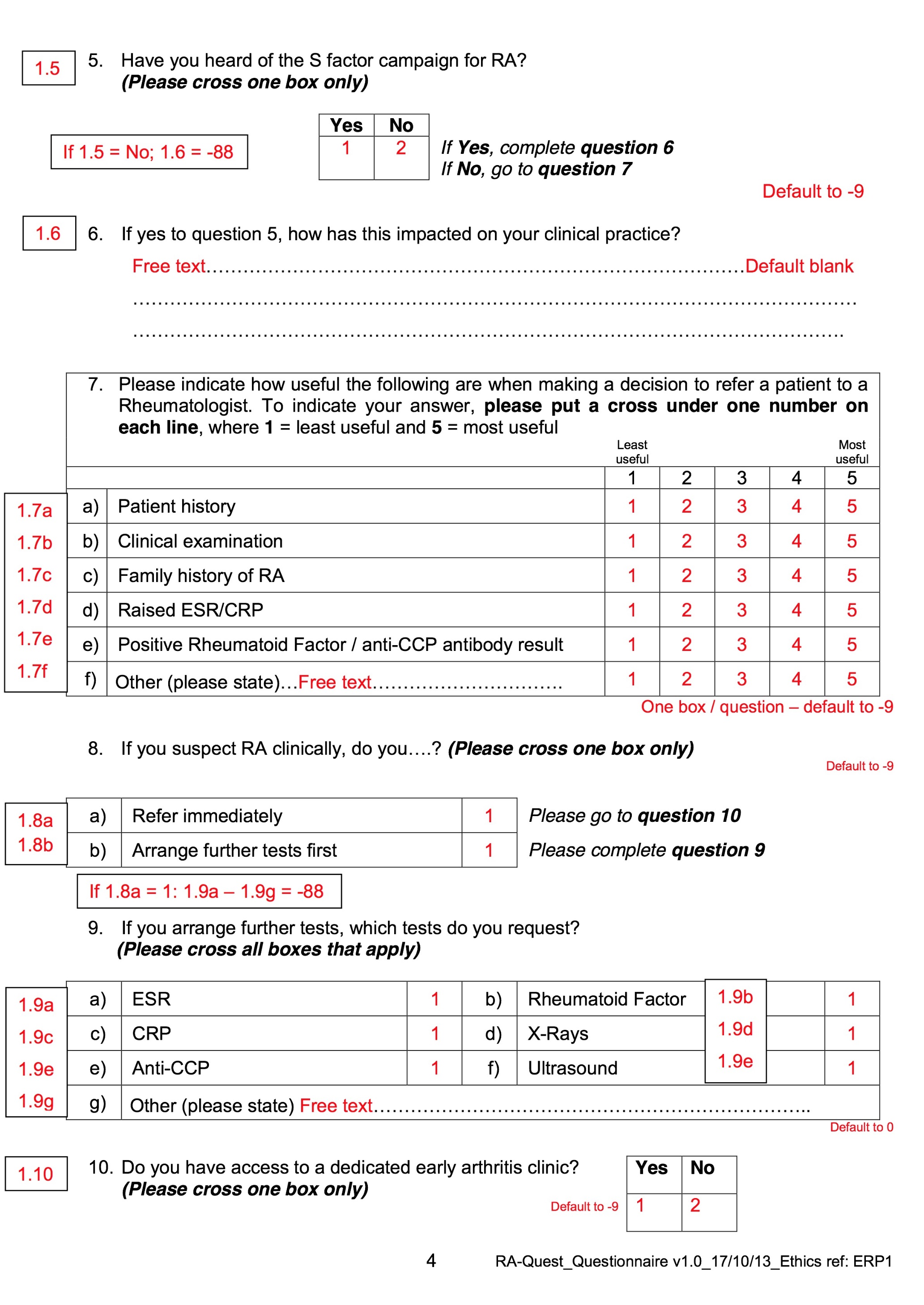
**

**
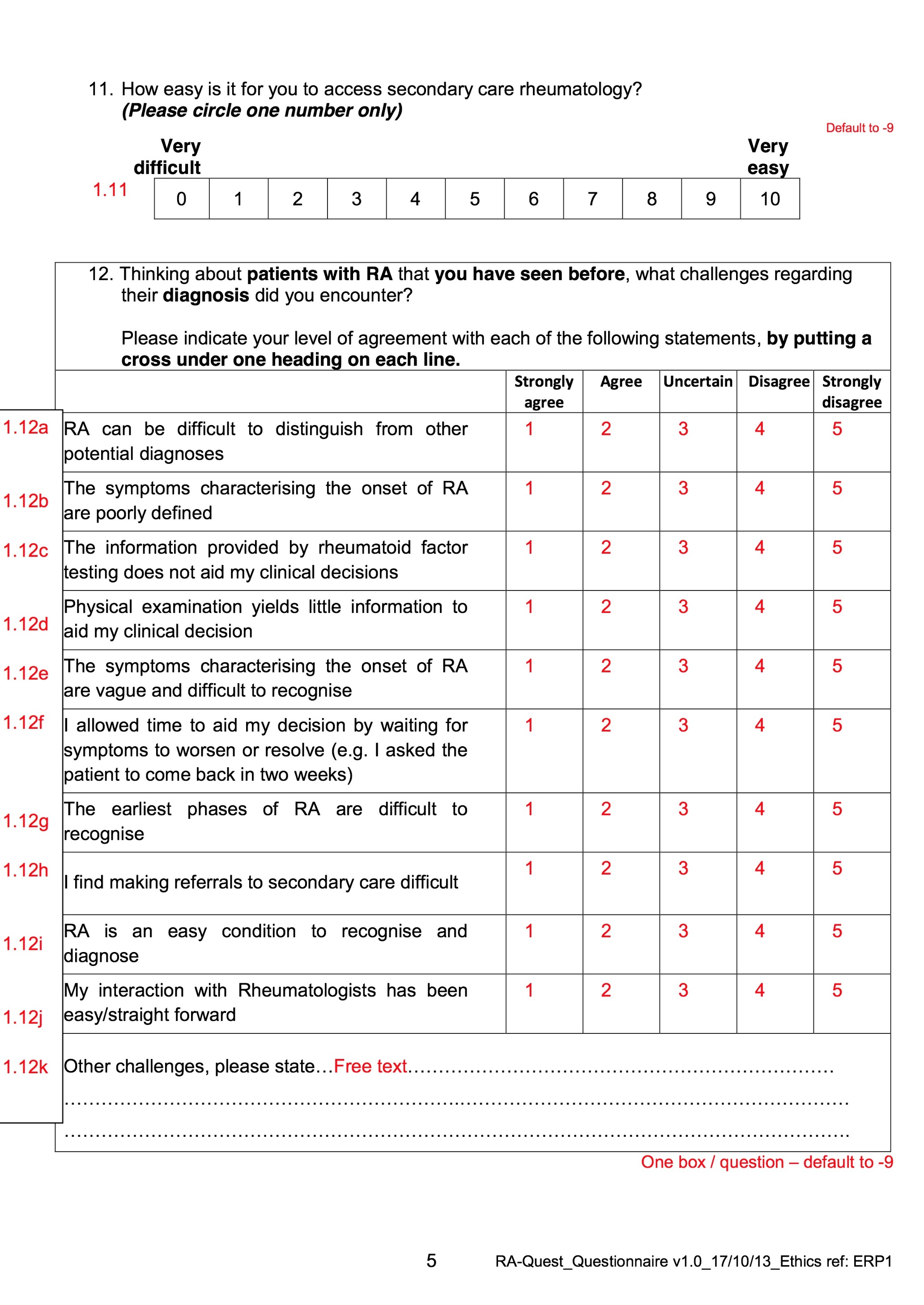
**

**
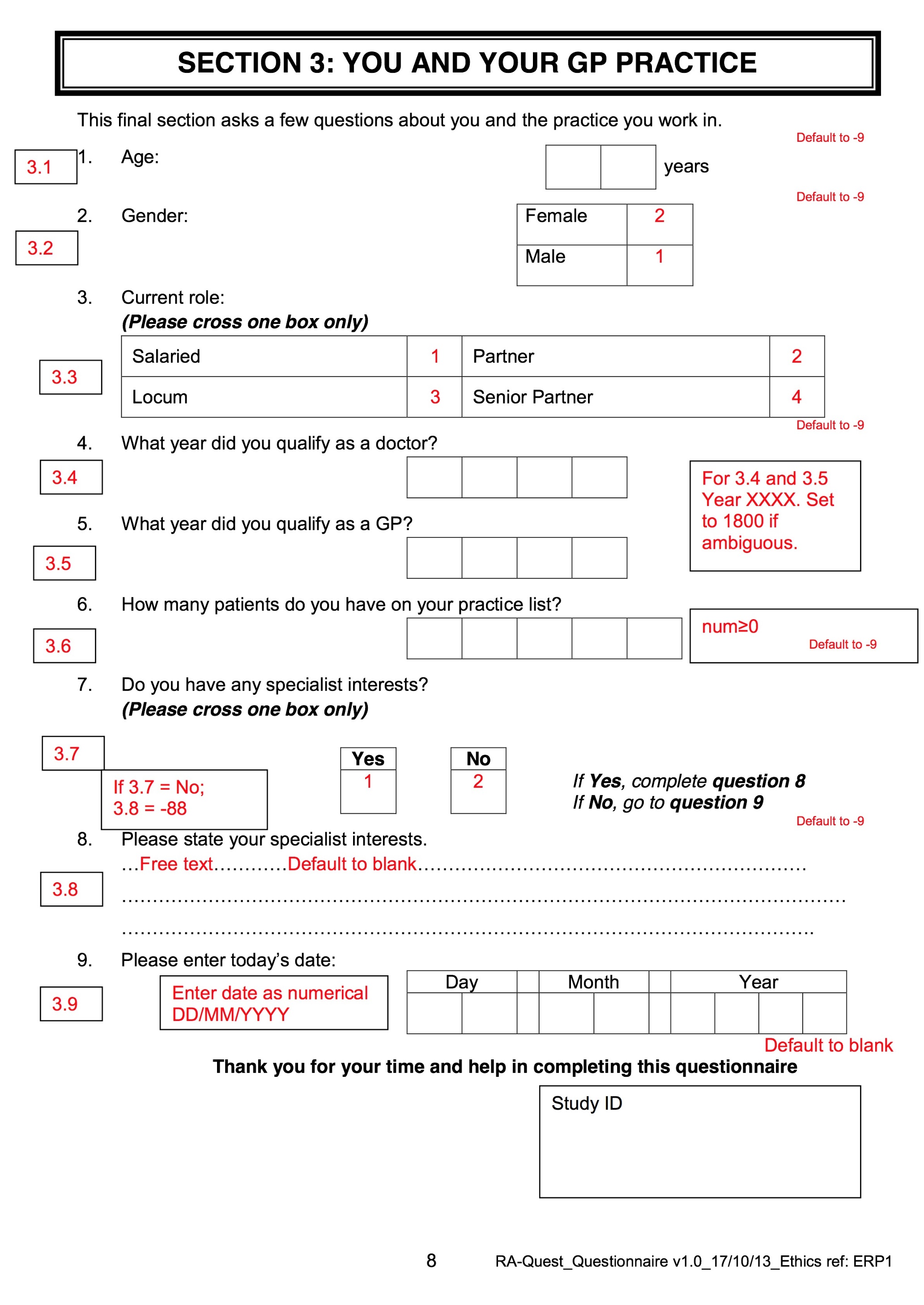
**

**Supplementary Figure 2. Free-Text Responses to Questions 6 (Impact of S-Factor Campaign on Clinical Practice), 7 (Useful Features in Making Referral Decisions), 9 (Tests Informing Referral Decisions), and 12 (Challenges in Diagnosing RA).**

**
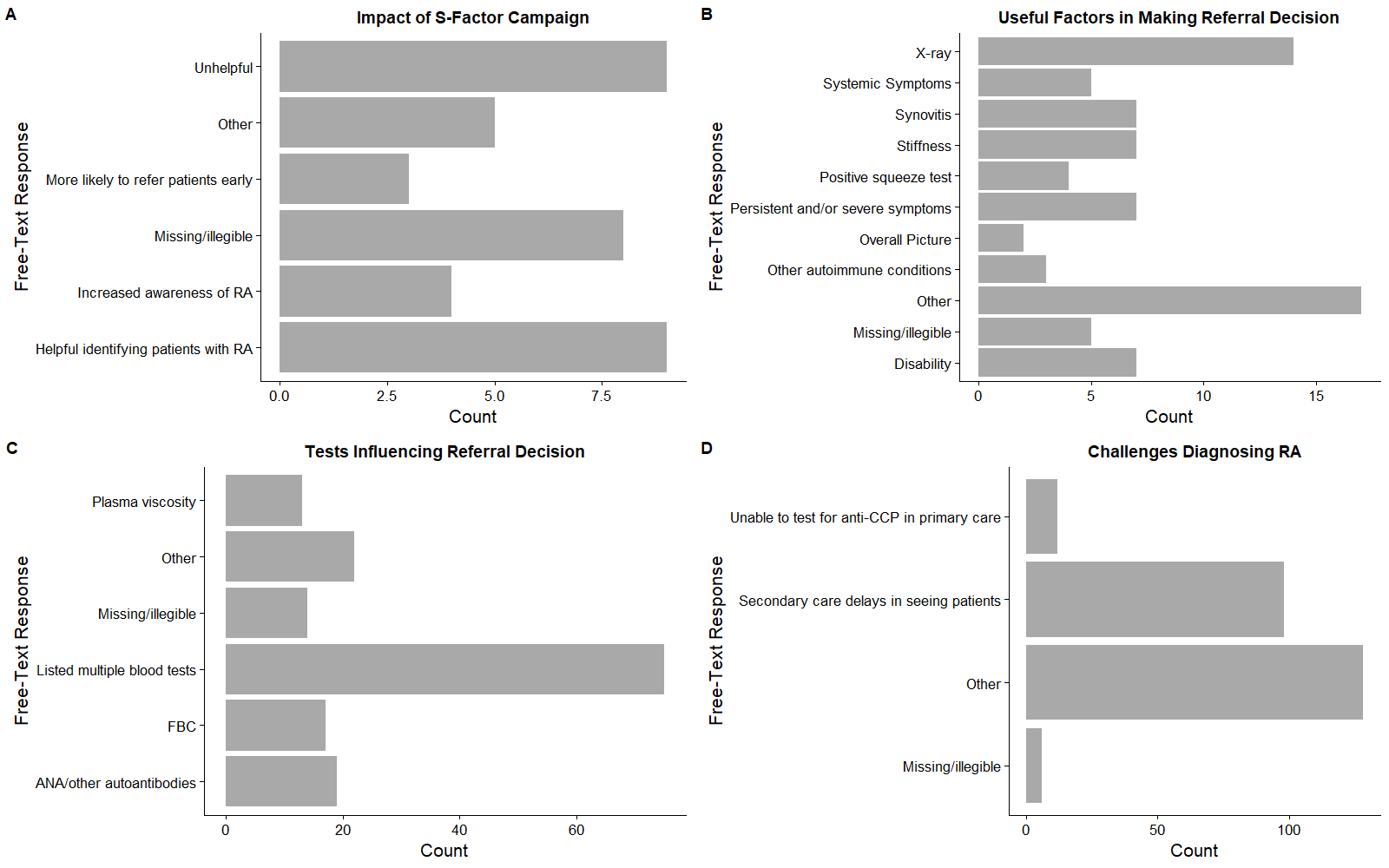
**

The graphs are “bar-plots” showing the number of individuals (count) providing free-text responses in each of the categories.

**Supplementary Figure 3. Likert Scales of Relative Importance of Different Clinical Features in Deciding to Refer a Patient with Possible RA (1 = Least Useful; 5 = Most Useful)**


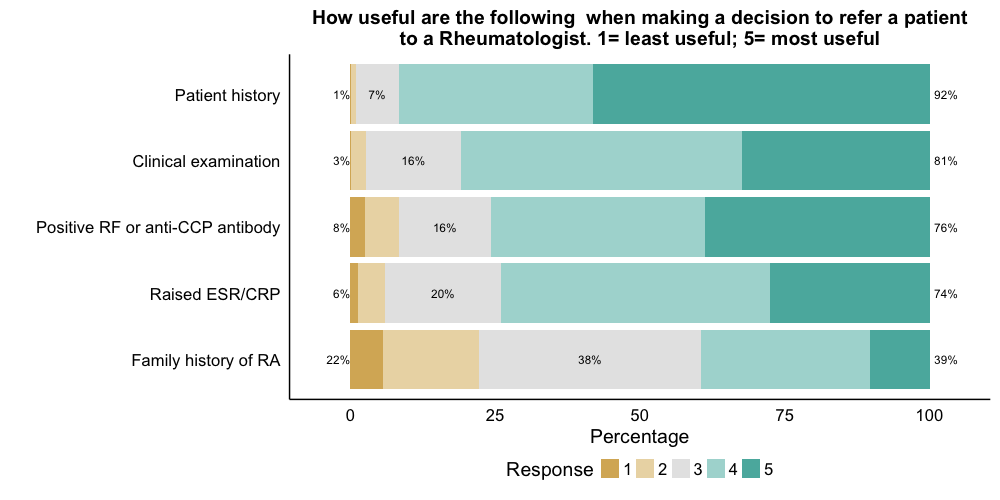

Supplement: Supplementary Data [file rky012_supp.docx]
